# Generating an Evidence Base for Information, Education and Communication Needs of the Community Regarding Deafness: A Qualitative Study

**DOI:** 10.4103/0970-0218.69275

**Published:** 2010-07

**Authors:** Neelima Gupta, Arun Sharma, PP Singh

**Affiliations:** Departments of Otorhinolaryngology India; 1 Community Medicine, University College of Medical Sciences and GTB Hospital, New Delhi, India

**Keywords:** Deafness, focus group discussions, IEC resources

## Abstract

**Background:**

India is a significant contributor to the world’s total burden of deafness. Out of all causes, almost 50% of the causes of decreased hearing are preventable. With the launch of the National Programme for Prevention and Control of Deafness, the need for an effective information, education and communication (IEC) campaign was felt. There is negligible information available about the status of awareness levels of the community about the various aspects of hearing loss. We carried out this research with the objective of getting to know the existing awareness related to hearing loss in the community to generate an evidence base for formulating various messages to be incorporated in IEC materials for dissemination in the community. We also asked the participants about their suggestions for the various information resources so that an IEC campaign could be designed accordingly.

**Materials and Methods:**

We carried out 10 focus group discussions among various groups of population and analyzed the discussion.

**Results:**

A descriptive analysis of the observations regarding the awareness about deafness in the community and prevalent myths and suggested information resources is presented.

**Conclusion:**

We highlight the lacunae in the existing awareness of various causes of deafness and the preventive measures that could be taken to prevent hearing loss. The evidence generated was used to formulate relevant messages for the various target groups, which were then incorporated in development of the IEC materials for the dissemination in the community.

## Introduction

The sense of hearing is a crucial faculty for the humans to communicate and thereby adapt in the society. At least 250 million people globally have hearing loss, i.e., more than 4% of the world’s population. At least two-thirds of these people, about 165 million, live in developing countries.(1)

It had been estimated in a study by UNICEF that nearly 80 million people suffered from hearing impairment of various grades in India. Out of these, 35 million were below the age of 14 years. More importantly, more than 50% of these disabilities were preventable.(2)

In a study coordinated by ICMR from 1986 to 1990, knowledge, attitude and practice (KAP) related to deafness were analyzed. The basic problems found in the rural areas for the lack of health awareness were poverty, illiteracy, ignorance, poor hygiene, inadequate medical guidance, and age-old customs and beliefs. Only 28% of the mothers and 51% of the teachers had correct information regarding ear care.(2) Training was given to health workers and teachers who in turn passed the knowledge to the experimental population. Primary and secondary prevention were stressed upon and at the end of the study there was a perceptible change in the hearing status of the population as against the control areas where inputs were not given. This created a sensitization for the need of effective IEC materials for the community and the involved personnel.

Recognizing the need for an effective IEC campaign for deafness, we decided to conduct a qualitative study in the community, to assess awareness related to causes and effects of hearing loss and practices regarding the treatment of deafness-related conditions so that we could formulate relevant messages to be disseminated in the community.

## Materials and Methods

Considering that there were various stakeholders, and information needed to be collected from diverse population groups, we used qualitative methods to examine the responses. After identifying the stakeholders, we used the focus group discussions (FGDs) as a tool for collecting information. Ten FGDs were conducted with 104 participants as per the break-up given in the Table 1.

**Table 1:** Focus group discussion breakup

Focus froup discussion	No. of participants
Junior doctors	10
Experts from relevant clinical departments	10
Multipurpose health workers/ANMs	11
Multipurpose health workers/ANMs	10
Patients of deafness visiting ENT department	12
Patients of deafness visiting ENT department	11
School teachers	10
Residents of a rehabilitation colony	10
Residents of a middle-income-group housing colony	10
Parents of children of a deaf and dumb school	10

The dual moderator focus group variant was utilized, where one moderator ensures the session progresses smoothly, while another ensures that all the topics are covered.

*Sampling of participants*: Sampling was done conveniently.

We broadly identified groups of various stakeholders, approached them, and held discussions with a group of participants who were willing to be a part of the discussion.

*Location of the study*: National Capital Region, New Delhi.

## Results

The salient points and the main observations derived from the discussions with each group are presented.

### Junior doctors

The participants said that hearing impairment is an important public health problem that has not been given due importance in medical education as well as by the general community. The role of loud music in clubs and excessive use of headphones for listening to music as a cause of hearing impairment was highlighted. All the participants were in favor of use of mass media like television and newspapers for creating awareness about deafness. They suggested that health workers should be trained in disseminating information about hearing impairment through community meetings.

### Clinicians

The doctors from different specializations opined that hearing loss as a presenting complaint was uncommon. According to pediatricians, mothers gave more emphasis to the problem of ear discharge rather than loss of hearing in their children. Elderly persons, according to ophthalmologists, were more concerned about loss of vision then loss of hearing. The commonly reported wrong practices in the community were putting mustard or garlic oil in the ear, indiscriminate use of ear buds, getting ears cleaned by roadside quacks, and wrongful use of eardrops.

The role of early diagnosis and treatment of upper respiratory infections, precautions regarding use of drugs during pregnancy, and awareness about damaging effects of noise were considered important aspects that should be covered in awareness campaigns. Some experts favored television over other media, while others felt that radio had more penetration then TV particularly in rural areas. Awareness generation among doctors was as important as in the general community.

### Health workers

Most of the auxiliary nurse midwives opined that they had been seeing about 5–10 persons with deafness in every village. Discharging ear was a very common problem in children, so much so that if the ear of a child was not discharging the mother would be worried that something is wrong with the ears of that child. They admitted their lack of awareness for methods of early diagnosis of decreased hearing in children. Usually they referred the patients to tertiary care hospitals, but acceptance of such advice was relatively poor in the community because of reasons like long queues in hospitals, loss of earning time for daily wage workers, and general indifference of hospital staff. The prevalent practices highlighted were putting oil in the ear or cleaning with objects like broom stick, matchstick, hair clip, etc. One startling observation of a participant was that mothers put breast milk in the infant’s ear as a treatment for deafness.

Methods suggested for creating awareness among the people were health talks with women folk in the village, with teachers and students in schools during health camps, and in village meetings that involve *panchayat* members and *anganwadi* workers.

### Patients attending OPD

These groups were of the opinion that there is a lack of awareness about where to seek treatment for diseases of the ear. Each one of them had known at least one person suffering from deafness, indicating a high prevalence of the problem. Practices enumerated were putting *neem* or garlic with mustard oil or snake oil in the ear, and assistance of *tantriks* and faith healers. However, majority of them agreed that such treatments were not effective in the people they knew. According to them, IEC messages could be similar to those for polio, TB, and AIDS with the involvement of celebrities for added impact. Holding meetings with groups of people and one-to-one communication may also be useful methods.

### School teachers

All the teachers were of the opinion that a national program for deafness prevention similar to the Blindness Prevention Program should be started. The only preventable cause of hearing loss according to them was noise-induced hearing loss. They were unaware of causes like ear infections and trauma. Methods suggested by them for awareness generation were conducting competitions among school children on slogan writing, poster making, essay writing, etc.; conducting health exhibitions; and lectures by health experts on special days like World Disability Day. Parent-teacher meetings were suggested as a good forum for talking about deafness, for sensitization of the parents. For the community, they suggested use of *nukkad nataks (street plays)*, screening of documentary films, and interactive sessions with deaf people. Writing articles in newspapers specially meant for children was another suggestion.

### Residents of a resettlement colony

They agreed that awareness is low in the community and efforts were needed to improve it. Ear infections and high-grade fever were quoted as causes leading to decreased hearing. They were not aware of preventive measures for ear diseases and hearing loss. They pointed out that people with decreased hearing gradually got excluded from social circles. Apart from the use of mass media like television and radio, other suggestions were use of hoardings and posters at public places like bus stops, markets and hospitals, and organizing film shows in parks.

### Residents of a group housing society

The participants cited ear infections, trauma, and any infection during pregnancy as causes of hearing loss. They were unaware that hitting on the ears, excessive listening to loud music through headphones, and indiscriminate use of ear buds could be harmful. As per them, it was important to provide services for diagnosis and treatment of ear diseases at dispensaries as well. They recommended dissemination of information in schools, institutions, and offices.

### Parents of children of deaf and dumb school

Most of the participants were unaware of the causes of deafness in spite of having a child with hearing impairment. Only two participants were aware that infection during pregnancy might cause hearing impairment in the newborn. Most of the participants expressed their inability to recognize the symptoms of deafness in small children. The earliest they could think of detecting hearing impairment was in a 1.5 to 2-year-old child. Prenatal counseling at antenatal clinics was suggested as a means of making prospective parents aware of the causes, prevention, and early diagnosis of hearing impairment in children. Putting up of display boards and hoardings at prominent sites in hospital premises, in addition to use of audiovisual mass media and handbills, were suggestions for an effective IEC campaign for deafness.

## Discussion

The importance of creating an IEC program for any community health problem cannot be overemphasized. IEC activities provide people the information they need to make informed choices about adopting and continuing healthy lifestyles.

Communication inputs increase people’s awareness and concern over matters relating to health and family welfare and subsequently decide to a large extent the limits of possible improvements and changes in these areas.(3) This prompted us to carry out this study in the community and collect relevant data. On review of the literature, no specific studies were found that had studied awareness in the community toward various aspects of hearing loss, the messages that were required to be communicated to the community, and methods that the various stakeholders would advocate for the education of the community at large.

In our qualitative study, the groups consisting of the ANMs were in our opinion the most well informed. However, they also expressed their inability to identify a child with suspected hearing loss. The FGDs brought out the issues like lack of awareness about preventable causes of deafness such as ear infections, congenital hearing loss, noise-induced hearing loss, drug-induced hearing loss, and hearing loss due to trauma. Various myths and prevalent practices such as putting mother’s milk, *neem* or garlic oil in the ear of a child and going to *tantriks* and faith healers for ear diseases were highlighted from discussion among patients attending OPD. Lack of awareness of importance of proper and timely vaccination for the prevention of hearing loss and lack of availability of good diagnostic and treatment facilities as well as rehabilitation facilities were also brought out. An evidence base was generated for the formulation and dissemination of messages such as, “not to get ears cleaned by road side quacks,” “not to hit anyone on the ears,” “avoid excessive exposure to loud sounds,” etc. The need for messages stressing the importance and effectiveness of rehabilitation of hearing loss was also brought out.

### Relevance of information, education and communication materials

Studies in the past have stressed the importance of education and communication in the prevention of deafness at one level or the other.

In a study by King *et al*.,(4) it was found that during 16,070 consultations in general practice, 70 adults complained of difficulty in hearing. A simple poster displayed in the waiting areas almost doubled the incidence of presentation of loss of hearing. The beneficial effect was lost almost as soon as the poster was removed, thus highlighting the effectiveness of a simple tool such as a poster.

In a German study, the statistics in 1981 showed the average age at initial diagnosis of children with hearing impairment to be between 3.3–3.4 years. In 1989, this average age came down to 13.4 months. This favorable result was attributed to the population being informed by special professional events for various target groups.(5)

Many studies have highlighted the importance of noise exposure as a preventable cause of deafness if effective IEC material is created and community is educated about the harmful effects of noise. Brookhouser *et al*.(6) in their study of noise-induced hearing loss found that NIHL in the pediatric population has received scant attention. This fact was also brought out in our discussion with teachers and junior doctors.

In a consensus conference on NIHL(7) it was said that hearing conservation must begin by providing each individual with basic information. Educational programs should be targeted toward children, parents, hobby groups, public role models, and professionals such as teachers, physicians, and educators. We conducted FGDs involving some of these groups to generate evidence for IEC needs in these groups which we have highlighted in our results.

After generating an evidence base for the IEC needs of the community, we propose following specific recommendations:

- The social and economic effects of deafness and its prevalence needs to be highlighted in the IEC materials.
- Awareness to be promoted in junior doctors and medical officers through CMEs and periodic updates.
- Training the ANMs for primary ear care and recognizing patients that require referral. Creating IEC materials and kits for the ANMs such as flip charts to help them in spreading information in the community for early diagnosis of hearing loss.
- Creating awareness in teachers through messages for the recognition of a child with hearing loss and to spread awareness in children about NIHL and preventing trauma to the ear.
- Importance of prenatal counseling and early screening for hearing loss in children to be stressed in messages for the antenatal clinics.
- Making information about treatment, diagnostic, and rehabilitation centers to be easily and widely available to the community through mass media.

## Conclusion

The need for disseminating information about various aspects of deafness was felt by all the groups. Awareness levels were low, wrong practices were prevalent in the community, and there was lack of serious concern for deafness as a health problem. Evidence was generated that messages needed to be formulated for primary ear care at home, early diagnosis of ear infections, screening of infants for suspected hearing loss, avoidance of high noise levels, antenatal counseling, and proper rehabilitation of people with decreased hearing. Evidence was also generated that it was felt that mass media would play a major role in an effective IEC campaign for deafness along with increased community participation through involvement of the grassroot primary health care workers.

## References

[1] Kumar S (2001). WHO tackles hearing disabilities in developing world. Lancet.

[2] Kumar S (1997). Deafness and its prevention--Indian scenario. Indian J Pediatr.

[3] Freimuth VS, Edgar T, Fitzpatrick MA (1993). Introduction: The role of communication in health promotion. Communic Res.

[4] King RL, Barry B, Brooks DN (1987). Effectiveness of publicity campaign encouraging earlier referral of hearing loss in adults. Br Med J (Clin Res Ed).

[5] Aust G, Foll U, Lutt B, Schaffrath R (1989). Hearing handicap in childhood--responsibilities of public health, early diagnosis. Offentl Gesundheitswes.

[6] Brookhouser PE, Worthington DW, Kelly WJ (1992). Noise-Induced Hearing Loss in Children. Laryngoscope.

[7] (1990). Noise and Hearing loss-Consensus Conference. JAMA.

